# A data process of human knee joint kinematics obtained by motion-capture measurement

**DOI:** 10.1186/s12911-021-01483-0

**Published:** 2021-04-08

**Authors:** Jian-ping Wang, Shi-hua Wang, Yan-qing Wang, Hai Hu, Jin-wei Yu, Xuan Zhao, Jin-lai Liu, Xu Chen, Yu Li

**Affiliations:** 1 School of Mechanical and Power Engineering, Henan Polytechnic University, Jiaozuo, 454000 China; 2 School of Medical Technology, Medical University, Qiqihar, 161006 Heilongjiang Province China; 3 Shanghai Sixth People’s Hospital, Shanghai, 200233 China; 4 The First Affiliated Hospital of Henan Polytechnic University, Jiaozuo, 454000 Henan Province China

**Keywords:** Knee joint, Motion capture measurement, MATLAB, Kinematics

## Abstract

**Background:**

The motion capture has been used as the usual method for measuring movement parameters of human, and most of the measuring data are obtained by partial manual process based on commercial software. An automatic kinematics data process was developed by programming on MATLAB software in this paper.

**Methods:**

The motion capture measurement of healthy volunteers was carried out and the MATLAB program was used for data process. Firstly, the coordinate data of markers and anatomical points on human lower limb measured by motion capture system were read and repaired through the usual and the patch program. Meantime, the local coordinate systems of human femur and tibia were established with anatomical points. Then flexion/extension, abduction/adduction and internal/external rotation of human knee tibiofemoral joint were obtained by special coordinate transformation program.

**Results:**

Using the above methods, motion capture measurements and batch data processing were carried out on squatting and climbing stairs of 29 healthy volunteers. And the motion characteristics (flexion/extension, internal/external rotation and adduction/abduction) of the knee joint were obtained. For example, the maximum internal/external rotation in squatting and climbing stairs were respectively was 30.5 degrees and 14 degrees, etc. Meantime, the results of this paper also were respectively compared with the results processed by other research methods, and the results were basically consistent, thus the reliability of our research method was verified. After calibration processing, the compiled MATLAB program of this paper can directly be used for efficient batch processing and avoiding manual modeling one by one.

**Conclusion:**

A novel Patch Program of this paper has been developed, which can make reasonable compensation for missing and noise signals to obtain more complete motion data. At the same time, a universal data processing program has also been developed for obtaining the relative movement of various components of the human body, and the program can be modified for detail special analysis. These motion capture technologies can be used to judge whether the human body functions are abnormal, provide a reference for rehabilitation treatment and design of rehabilitation equipment, and evaluate the effectiveness before and after surgery.

## Background

Total Knee Arthroplasty (TKA) is a routine operation used to treat knee-related diseases in humans [1–3]. By measuring the lower limbs of healthy volunteers of a certain species, the anatomical characteristics and movement parameters of the knee were obtained, which can provide reference for the design and optimization of artificial knee prosthesis in line with the characteristics of this species. The gait measurement is used to find out whether there is a diseased condition in the human body and compare the motion parameters before and after the operation for analyzing the treatment effect of the operation and further formulating the treatment plan [4]. With the development of motion capture technology [5–7], some scholars carry out motion capture measurement of human gait [8–10], some scholars have made motion capture measurement and analysis of human knee joint squatting [11, 12], and some scholars use the motion capture system to measure and analyze the motion characteristics of the lower extremity joints during the stair climbing process [13–16]. Many of them use the commercial motion capture systems such as Visual3D (C-Motion, USA) and Vicon (Oxford Metrics Limited, UK) to calculate and analyze the kinematic parameters of the human knee joint; these methods need to be manually processed one by one for modeling, which are time-consuming and labor-intensive [17]. For a large amount of data, it is necessary to effectively deal with missing values, normalize data sets and seek efficient processing methods [18–20]. One of the challenges in estimation missing value methods is how to select the optimal number of nearest neighbors of those values [21–23]. And it is becoming ever more critical to efficiently choose the most optimum machine or method on which to execute a program [24, 25]. Data analysis and prediction can be regarded as an effective method [26, 27]. However, by using MATLAB (MathWorks, USA), according to the preparation, calibration, measurement process and characteristics of motion capture measurement, a special program for motion capture measurement can be written [28].

In this paper, a motion capture data process method was developed to measure kinematic characters parameters in squatting and stairs-climbing of healthy volunteers. After calibration processing, the compiled MATLAB program in this paper can directly be used for efficient batch processing avoiding manual modeling one by one, which is convenient and fast. A novel Patch Program has been developed to make reasonable compensation for missing and noise signals to obtain more complete motion data. Meanwhile, a universal data processing program has also been developed for obtaining the relative movement of various components of the human body, and the program can be modified for detail analysis. And it is specially useful to study prosthesis and rehabilitation of patients after total knee arthroplasty.

## Methods

### Subjects

There were 29 healthy experimental volunteers, including 17 males and 12 females within 150–178 cm height (average 165.5 cm) and 47–81 kg weight (average 63.9 kg). The population of Shanghai urban area was selected as volunteers: 15 young volunteers aged 22–28 years old and 14 middle-aged volunteers aged 41–71 years old.

### Instrumentation

**Fig. 1:**
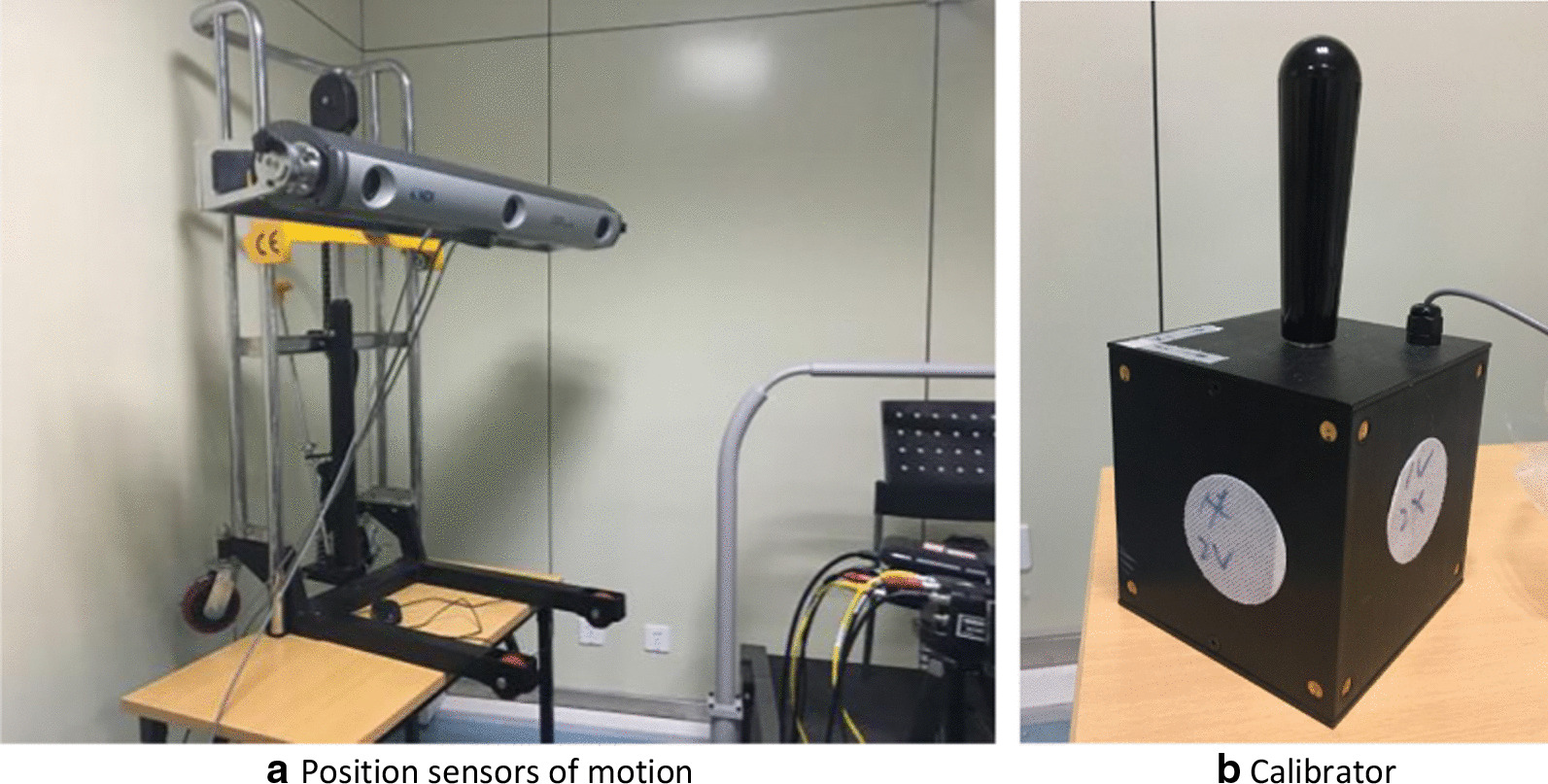
The Optotrak Certus motion capture system. *Note*: The Optotrak Certus (NDI) Motion Capture System was from Canada. Accuracy: 0.1 mm; resolution: 0.01 mm; maximum sampling frequency: 2000 Hz; mark point frequency: 4600 Hz; the maximum support marker (number): 512; the minimum marker diameter: 4 mm

The experiment of motion capture measurement was carried out in Shanghai Sixth People's hospital. The Optotrak Certus (NDI, Canada) motion capture system with two position sensors were used, as shown in Fig. 1a.

### The experimental process

**Fig. 2:**
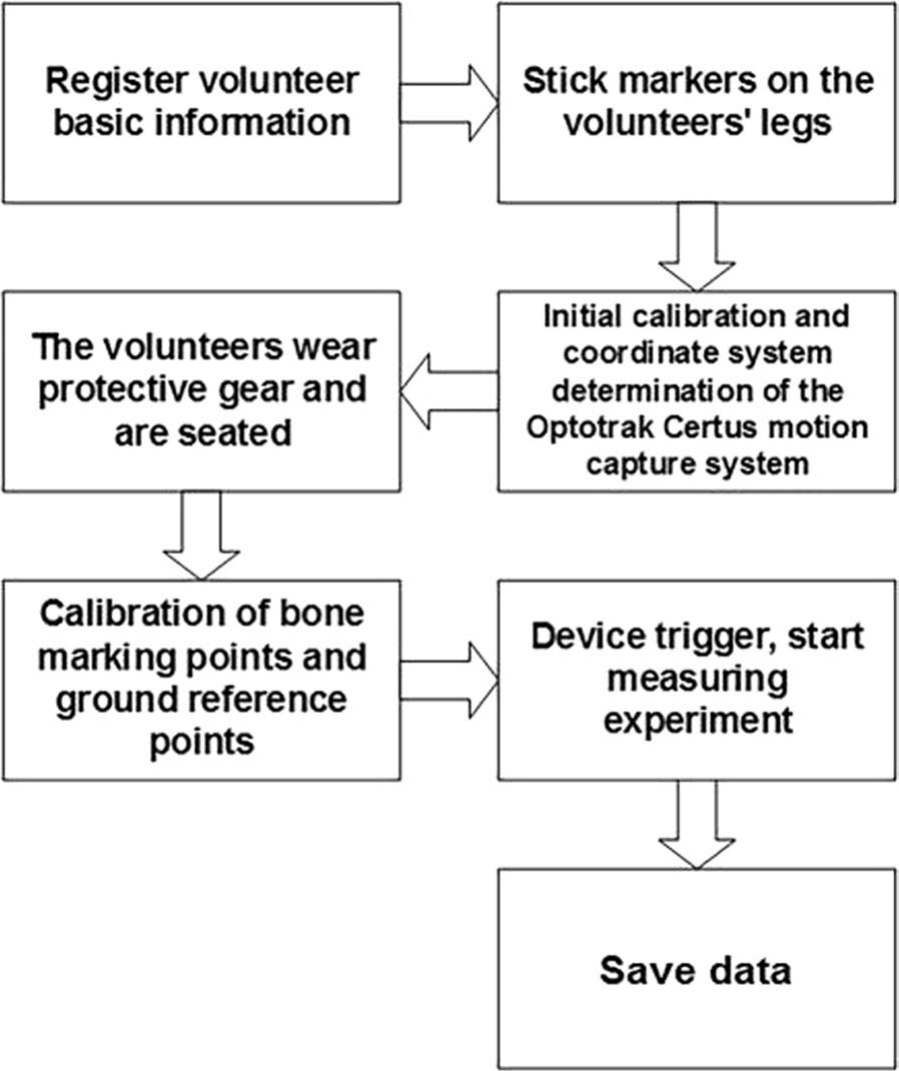
Motion capture measurement flow chart

In this experiment, the right leg movement of volunteers was measured. Then the calibration of the motion capture was performed sequentially; it includes: (1) Initial Calibration (the calibrator (as shown in Fig. 1b) was swung and rotated for 15 s in the common area of the two NDI position sensors to locate the two NDI lenses.) (2) Determination of the coordinate system (the three points on the motion capture device were taken to determine the x-axis, Y-axis and z-axis, so as to define the coordinate system of the motion capture system.) (3) The calibration of the seven bone markers (the probe was used to calibrate the seven bony markers for determining the coordinate system of the femur and tibia) and the ground reference point. The seven bone markers are respectively as following: the femoral trochanter, the femoral medial–lateral condyle, the medial–lateral tibial plateau, and the medial–lateral condyles of ankle joints. The detailed processes, from registering volunteer information and calibrating to finally saving data, are shown in Fig. 2.

**Fig. 3:**
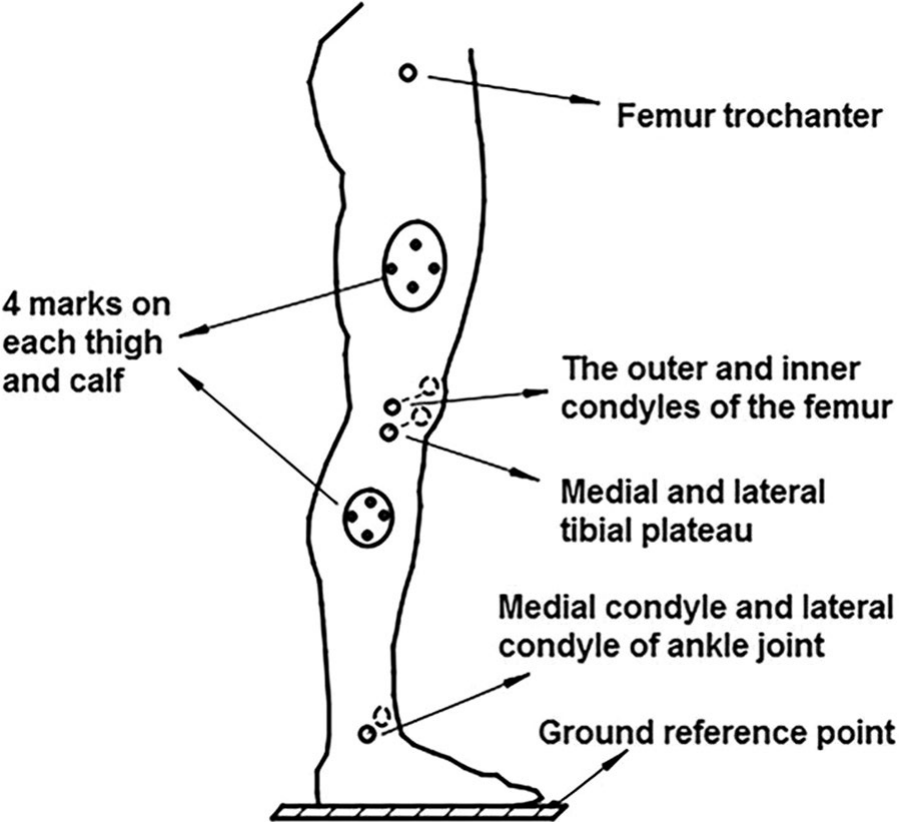
The position of markers and anatomic feature points in human lower extremity

As shown in the Fig. 3, there are 4 markers placed respectively on the thigh and calf, which were used for motion capture; meantime there are 7 anatomical feature points on the lower limbs and 1 ground reference point, which were used for calibration to determine the coordinate systems. In order to avoid the measurement error caused by skin movement or the bony point being occluded during the movements, virtual markers were generally used for calibration measurement [29], these 7 anatomical feature bony points are virtual markers for measurement. The coordinates of virtual-markers in the experiment can be determined by the coordinates of 3 or 4 marker points photographed on the rigid body. Therefore, the real-time coordinates of anatomical feature points of human lower limbs in the movement can be obtained by measuring the coordinates of markers pasted on volunteers' thighs and calves.

**Fig. 4:**
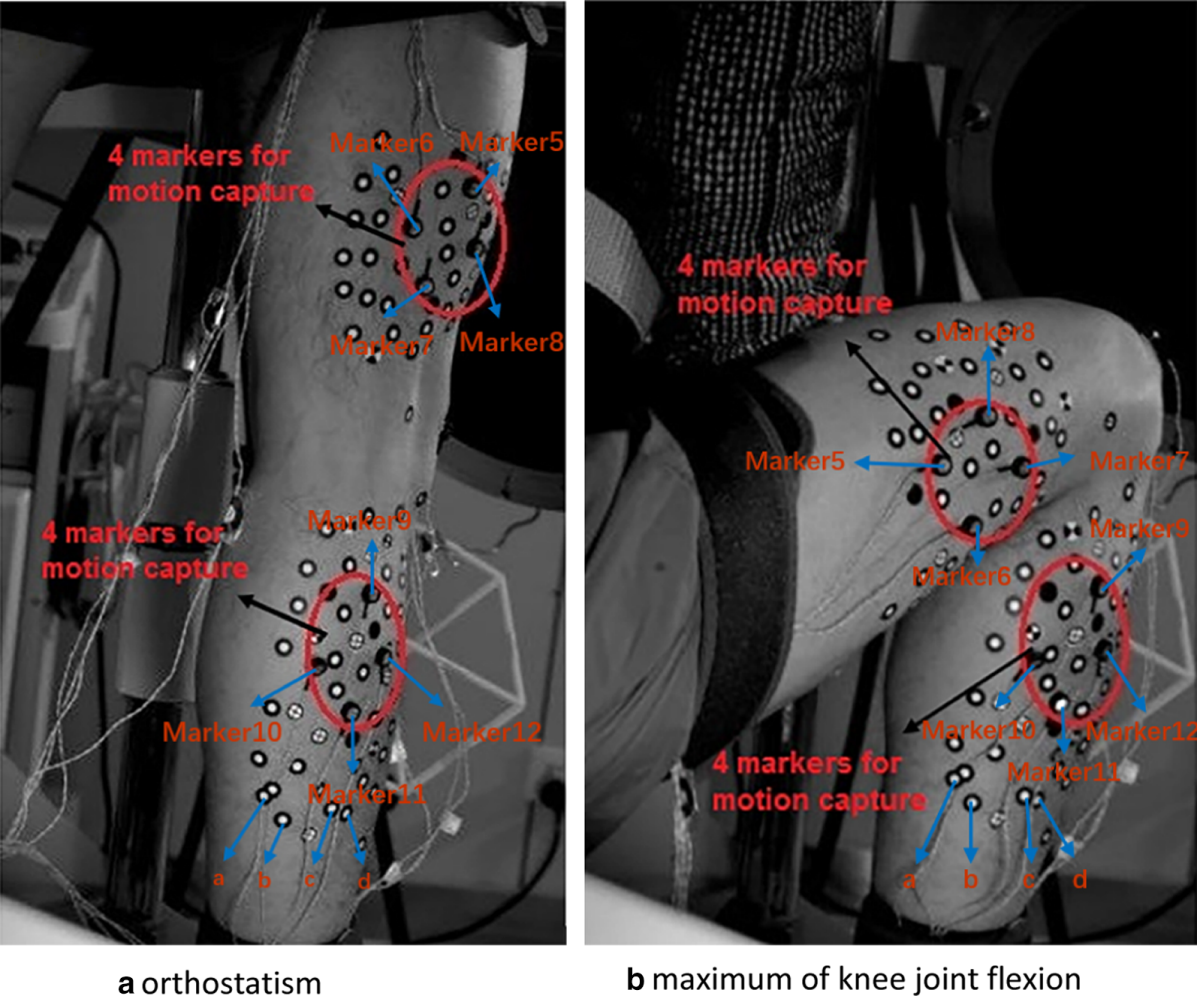
Squat movement measurement scene. *Note*: The picture shows a subject in our experiment, and the Optotrak Certus (NDI, Canada) motion capture system was used. The markers in black and white, such as the a–d markers of the calf, are used for other measurements of motion (RSA and HSSA) at the same time in this experiment

Spatial 3D (three-dimensional) coordinate data of marker points and bony points were saved by the motion capture system in a file in xls format. The motion capture measurement of the knee joint squatting are shown in Fig. 4, in which the Marker 5–8 on thigh and Marker 9–12 on calf were used to obtain the motion data. Figure 4a shows the initial squat measurement position when the lower limbs remain upright. Figure 4b is the position with the largest knee flexion angle in the squat. The rest markers in black and white, such as the a, b, c, d markers of the calf, are used for other measurements of motion at the same time in this experiment (RSA (Roentgen Stereophotogrammetric Analysis) and HSSA (high spped stereo radiography)), just for the further research.

### Data processing method

**Fig. 5:**
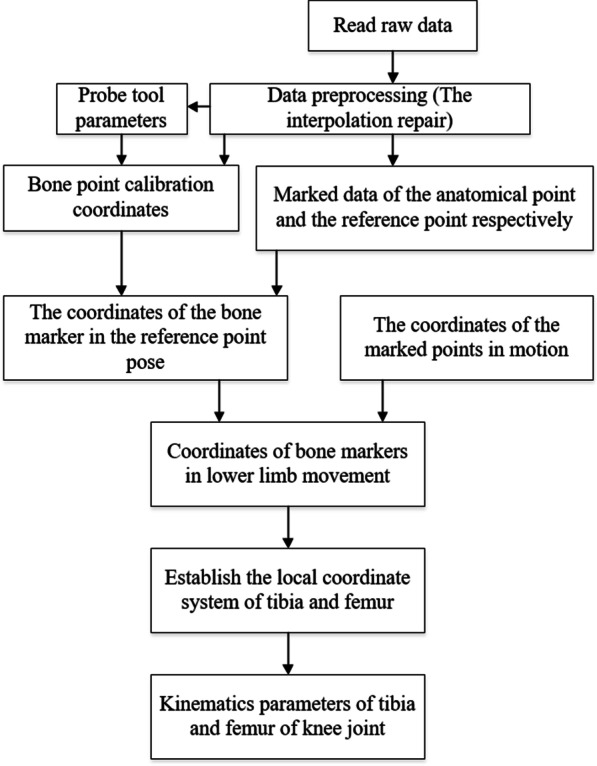
Flow chart of data processing method based on MATLAB. *Note*: The data processing program made by MATLAB was developed in this paper to obtain and analyze the 3D coordinate data of markers, from ‘reading raw data’ to ‘kinematics parameters of tibia and femur of knee joint’ shown in above

**Fig. 6:**
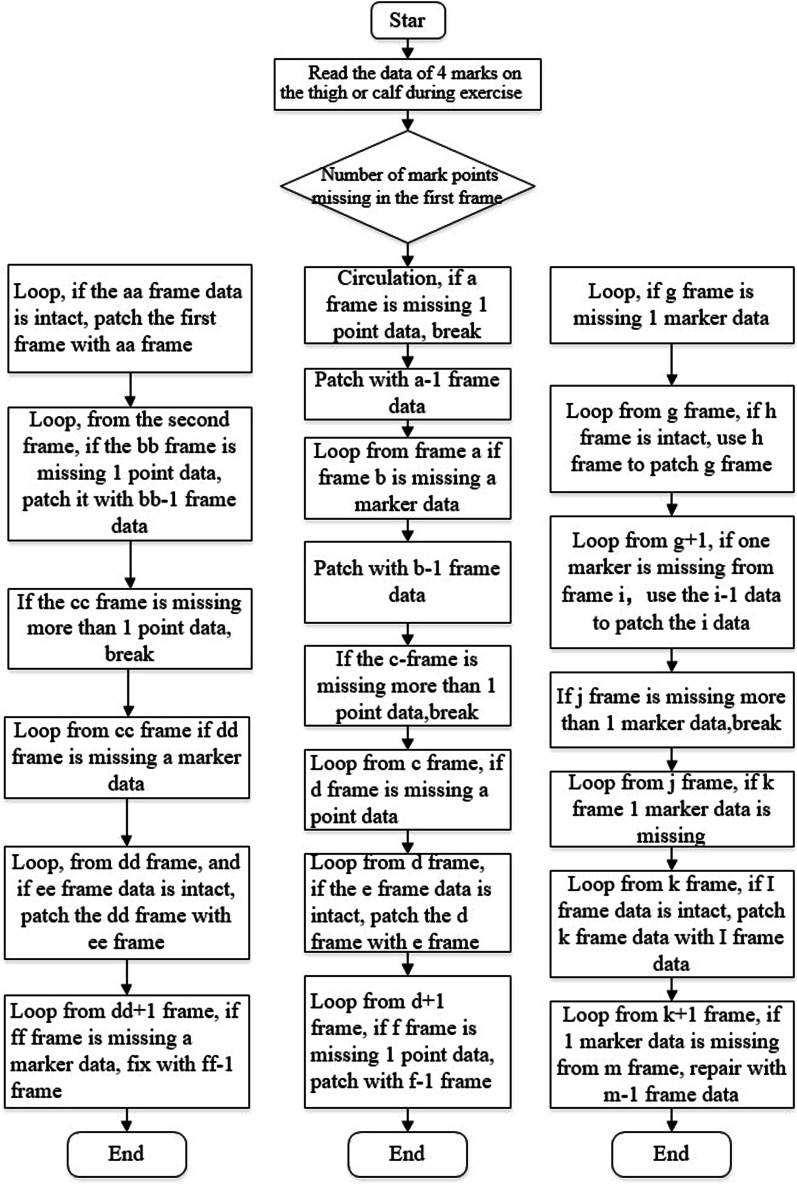
Patch Program flow chart. *Note*: This figure is the patching process flow chart of Patch Program developed by this author

Then, the data processing program made by MATLAB software was developed in this paper to obtain and analyze the 3D coordinate data of markers, as shown in Fig. 5. If the data was intact, it could be read and processed directly. Just the acquired data of markers 1–12 and 7 anatomical feature points (Fig. 3) needed to be read according to the program, so as to edit and obtain the motion tracks of multiple volunteers. And the process of data post-processing described in Fig. 5 is quick and convenient because of insteading of manually establishing marker one by one on the model in the post-processing of data in business software. At the same time, the coordinate system of each volunteer's femur and tibia can be automatically constructed according to the program in this paper. Then, a data processing program also has been developed in this paper for the relative movement analysis of various components of the human body, to obtain the kinematic parameters, for example, flexion/extension, adduction/abduction, and internal/external rotation of the knee tibiofemoral joint. If the collected data were missing or there were unnormal offsets, the original data should be firstly interpolated and repaired reasonably before further data process by the Patch Program shown in Fig. 6.

The written patch flowchart is shown in Fig. 6 below. In the experiment, there are 4 markers attached to the thighs and calves of the volunteers respectively. If there is 1 marker data missing in the Nth frame in the motion measurement, then according to the data of 4 markers in the previous frame or the nearest frame and the remaining three markers data of the Nth frame, the transformation matrix between the two sets of coordinates were established, thereby the coordinate data of missing markers in the Nth frame were solved.

### Reading and preprocessing of the original coordinate data of the marker points

The original data of experiment include: the coordinates of four markers (Marker 1–4) on the probe tool in the process of static state calibration; and the coordinates of eight markers (Markers 5–8 on the thigh and Markers 9–12 on the calf) respectively during the static state and movement. The original data of these markers were saved in xls files after motion capture test.

**Fig. 7:**
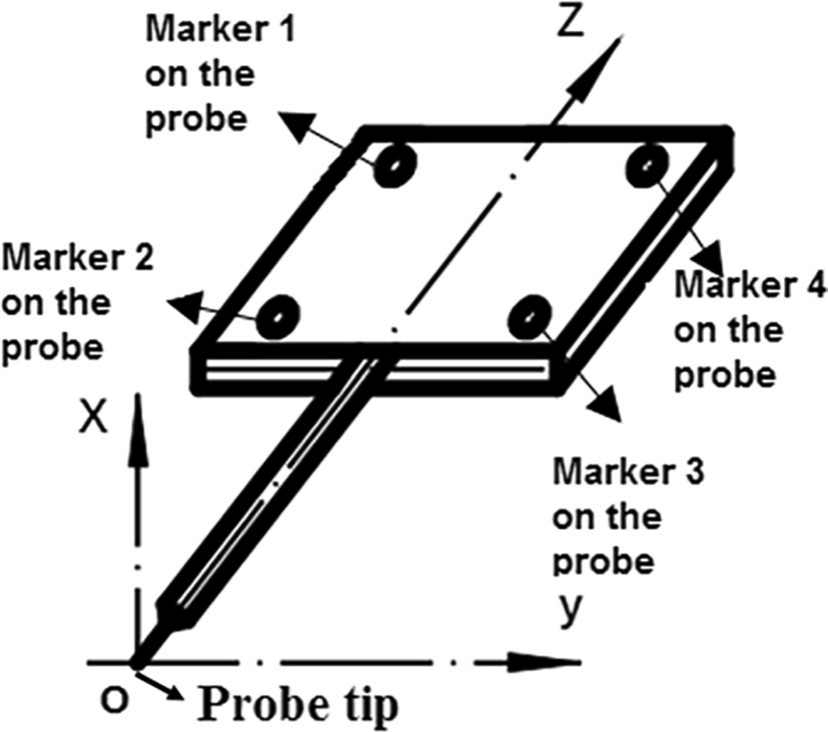
The probe and its local coordinate system

Firstly, the probe was used to calibrate the seven bony markers for determining the coordinate system of the femur and tibia. Figure 7 shows the schematic diagram of the probe and the local coordinate system of the probe, with the probe tip as the origin of the local coordinate system.

**Fig. 8:**
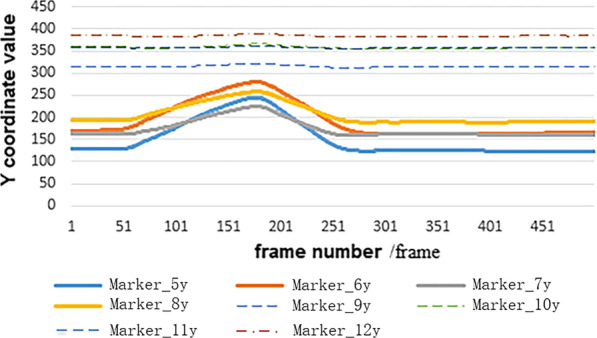
The coordinate values in Y direction of markers in thigh and calf under squatting. *Note*: Markers 5–8 are on the upper thigh and Markers 9–12 are on the calf. In addition, the X-axis and Z-axis directions change circles also can be obtained

And then, the data read function ‘xlsread’ of MATLAB was used to read coordinate data, one group of which (as an example) were figured and shown in Fig. 8.

Figure 8 shows the change of the coordinate value of the marker points on the squat legs in the up and down direction (Y coordinate direction). The horizontal coordinate represents the number of frames (500 frames, 100 frames per second). Markers 5–8 are on the thigh, markers 9–12 are on the lower leg, and the data of markers on the calf has a small change because of the small amplitude of the calf in the movement. It could be seen that in the first 50 frames, the data remains unchanged (the volunteers do not move), and then the squatting action begins. The data appears to be increasing firstly and then decreasing while the knee squatting.

In the experimental process, some of the coordinate data of markers were missing due to some of them being obscured. For the Integrity of action or motion characteristics, one patch program was developed in MATLAB based on the number of missing data and the missing position, to repair the missing data. As shown in Fig. 8, it takes 2.5 s for 50–300 frames to complete the collection from squatting to standing, in which the data-loss will be caused by marker points being obscured or falling during the movement. When one of the 4 markers points (which were on the thigh or calf, as shown in Fig. 3) missed, it can be repaired. However, it cannot be repaired and should be discarded when 2 or more markers of the 4 markers missed.

The positional correlation among markers during the movement and the overall movement trend are all references for data repair. For example, this relation can be obtained according to the real-time coordinates of the upright position and the maximum flexion position shown in Fig. 4a, b respectively, and the real-time coordinates of the anatomical feature points of the upright position were shown in Fig. 3. When the Patch Program was written, cubic spline interpolation function was used for interpolation repair [30]. The curve after interpolation by this method is second-order continuous and differentiable, with high precision and smooth curve transition. The detailed introduction of cubic spline interpolation function is described in literature [30], and the principle is shown in “Appendix 1”.

### Solution of coordinate data of bone markers in lower limb movement

According to the coordinate data of 4 marker points on the probe obtained by measurement and the parameters of the probe itself (coordinate data of 4 mark points in local coordinate system), then the transformation matrix and translation matrix between two sets of coordinate data was established, by which the coordinate data Z (probe-tip’s coordinates) of the bony-markers (also known as anatomical feature points) was obtained. According to the coordinate data of the marker points on the thigh and calf respectively in the calibration of bony-markers and ground reference points, the transformation matrix and the translation matrix between these two sets of coordinate data was established, which was used to calculate the coordinate data of bony-markers points in the calibration of ground reference points. Finally, according to the coordinate data of marker point on the leg in the calibration of ground reference point and the coordinate data during the movement of human lower limb, the transformation matrix between the two sets of coordinate data was established, by which the coordinate data of the bony-markers in the lower limb was obtained. The detailed introduction of solving the transformation matrix is described in literature [31], and the principle is shown in “Appendix 2”.

### Establishment of local coordinate system of femur and tibia

**Fig. 9:**
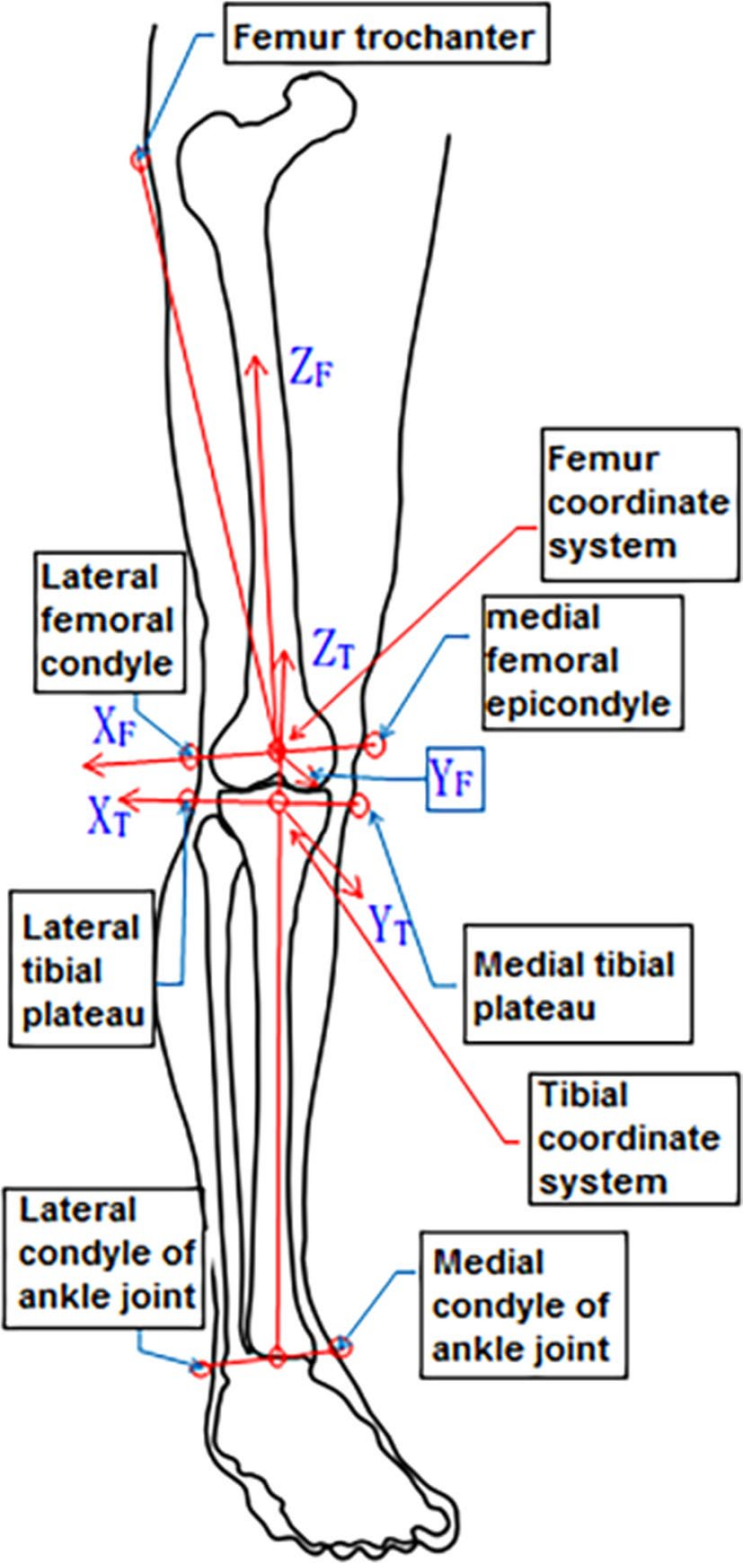
Knee joint femur and tibia local coordinate system**.** *Note*: X_F_, Y_F_, Z_F_ indicated the X-axis, Y-axis, Z-axis coordinate system of the femur. X_T_, Y_T_, Z_T_ indicated the X-axis, Y-axis, Z-axis coordinate system of the tibia

Based on the seven bony-markers of the lower extremities, the local coordinate system of the femur and tibia were established according to the combination of several below scholars' researches: in 1983, Grood et al. [32] first introduced the method of establishing the joint coordinate system for the 3D motion of joints. Later, other scholars redefined the method of establishing the local coordinate system of various parts of the human body [33–37]. Dabirrahmani [38] modified the coordinate system definition method proposed by Grood et al. [32]. Of course, some scholars have proposed the definition method of the coordinate system of human lower limbs and knee joints [2], which are shown in Fig. 9. The method is as follows:

First, a bullet list is as follows: X_F_, Y_F_, Z_F_ indicated the X-axis, Y-axis, Z-axis coordinate system of the femur. X_T_, Y_T_, Z_T_ indicated the X-axis, Y-axis, Z-axis coordinate system of the tibia. The local coordinate system of the femur: the line connecting the lateral femoral condyle and medial femoral epicondyle is the X_F_, the direction is pointing to the outside, and the midpoint of the line is the origin. The X_F_ multiplied by the line being connected the femur greater trochanter and the origin to obtain the Y_F_ with the direction facing forward. The X_F_ multiplied by the Y_F_ to obtain the Z_F_ with the direction facing up.

The local coordinate system of the tibia: the connection between the medial and lateral tibial plateau is the X_T_, the direction is pointing to the outside, the midpoint of the line is the origin, and the X_T_ multiplied by the line being connected the origin and the midpoint of the inner and outer ridges to obtain the Y_T_, and the direction is forward. The X_T_ multiplied by the Y_T_ to obtain the Z_T_ with the direction facing up.

### Solving knee kinematic parameters

According to the above method, the local coordinate systems of femur and tibia were established according to the bony-markers [39]. The femoral coordinate system was rotated and translated to obtain the tibia coordinate system. According to the order of coordinate rotation, coordinates transform can be divided into two categories [40]. One type is that the three axises were different for the three rotation transforms to rotate, the other type is that the first and third rotation axis are the same one, and these two types all have six rotation transformation orders. In this paper, the order of X–Y-Z is used for Euler rotation, and its transformation matrix is R:

1 $$R = \left[ {\begin{array}{*{20}c} {R_{11} } & {R_{12} } & {R_{13} } \\ {R_{21} } & {R_{22} } & {R_{23} } \\ {R_{31} } & {R_{32} } & {R_{33} } \\ \end{array} } \right]$$

The femoral coordinate system was orderly rotated α angle around the X axis, rotated β angle around the Y axis, rotated γ angle around the Z axis, and coincided with the tibia coordinate system. According to the coordinate transformation principle, the rotation transformation matrix R is as follows:

2 $$\begin{aligned} & \left\{ \begin{gathered} \alpha = a\tan 2\left( { - R_{32} ,R_{33} } \right) \hfill \\ \beta = a\tan 2\left( {R_{31} ,\sqrt {R_{11}^{2} + R_{12}^{2} } } \right) \hfill \\ \gamma = a\tan 2\left( { - R_{21} ,R_{11} } \right) \hfill \\ \end{gathered} \right. \\ & a\tan 2\left( {{\text{x}},{\text{y}}} \right) = a\tan ({\text{x}}/{\text{y}}) \\ \end{aligned}$$

**Fig. 10:**
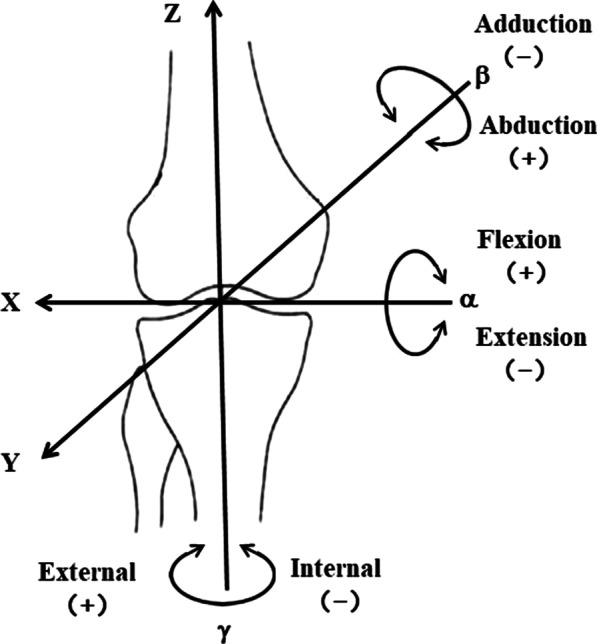
Joint angles are defined by rotations occurring about the three joint coordinate axes

According to the local coordinate system, the following definitions are made: the flexion/ extension angle of the femur relative to the tibia is the angle α of the femoral coordinate system rotating around the X axis, and the adduction/abduction angle is the angle β of the femoral coordinate system rotating around the Y axis. The internal/external rotation angle is the angle γ of the femoral coordinate system rotating around the Z axis, as shown in Fig. 10 below. The kinematic parameters of the knee joint during the lower limb movement are obtained by calculating the α, β and γ angle [32].

**Fig. 11:**
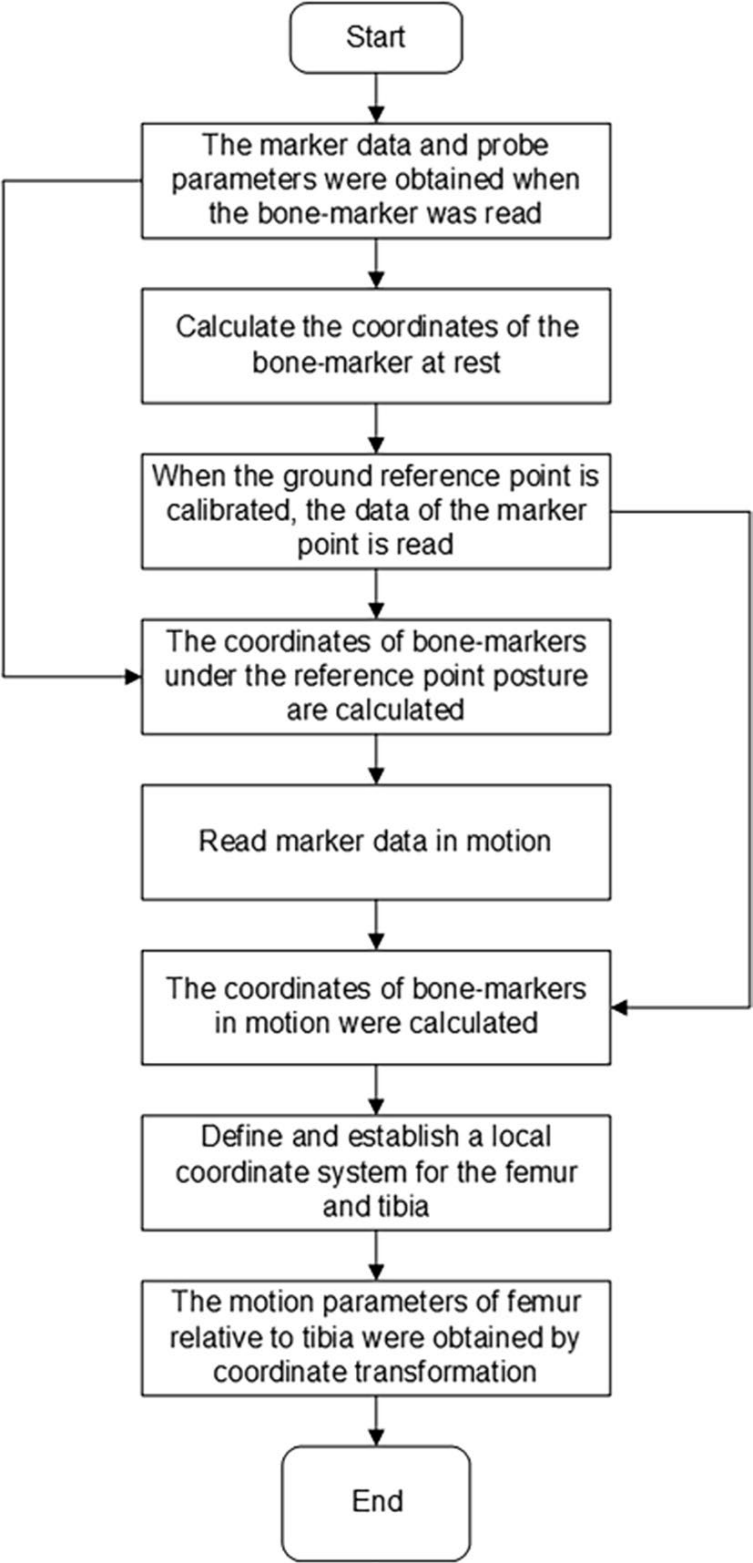
Main program flow chart of obtaining relative motion parameters of femorotibial joint

A universal data processing program has also been developed in this paper, for obtaining the relative movement of various components of the human body, and the program can be modified for detail analysis. Figure 11 is the flow chart of the main program for obtaining relative motion parameters of tibiofemoral joint, which were programed in MATLAB. The program starts reading the data by calculating the coordinates of the bony-markers firstly. Secondly, after the reference-points were calibrated, marker data is read and the coordinates of the bony-markers under the reference-points posture were calculated, then the marker data in the motion is read to calculate the coordinates of bony-markers in the movement; meantime, the local coordinate system of the femur and tibia were established according to the bony-markers. Finally the coordinate transformation was used to solve the kinematic parameters of the knee joint.

## Result

**Fig. 12:**
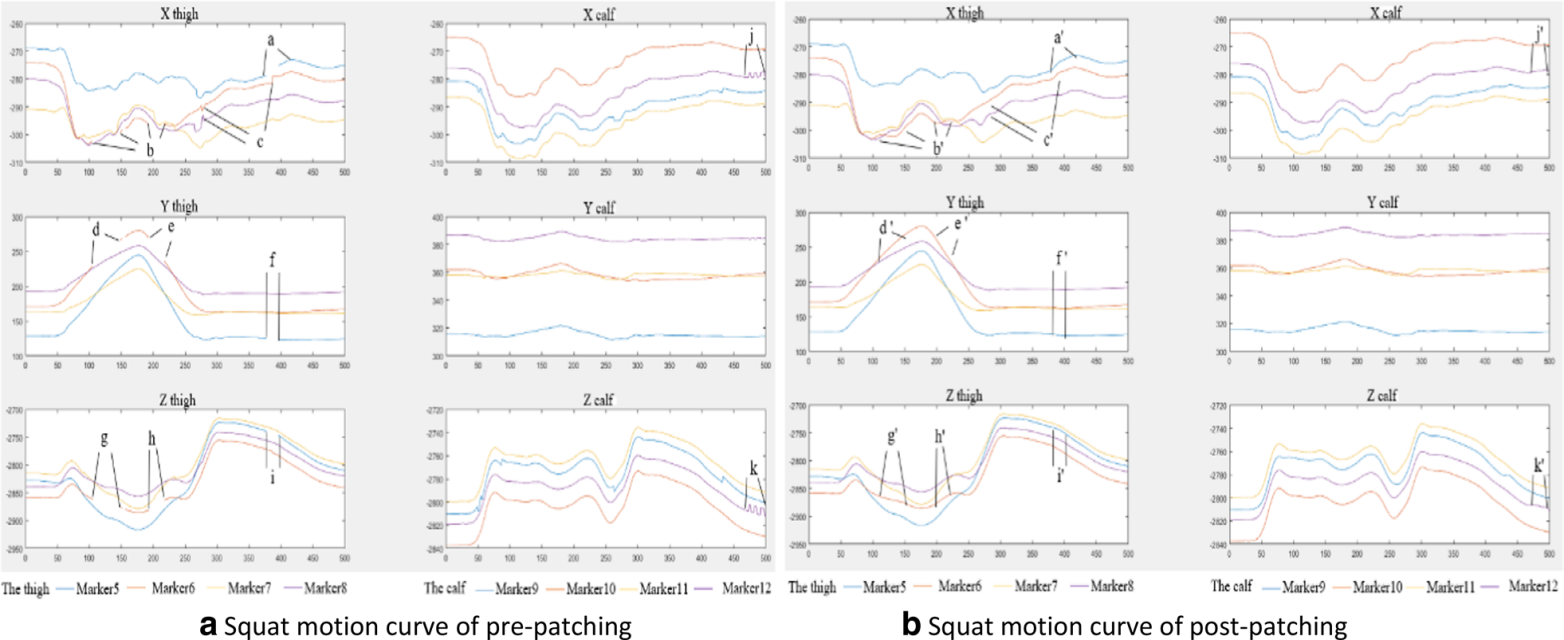
Pre-/post-patching motion curves of thighs and calves in squatting. *Note*: The X-axis represented the number of frames (500 frames) taken for each action in this experiment. The Y-axis represents the spatial coordinates of marker in Optotrak Certus system. The data missed at location a, b, d, e, f, g, h, i, and the data bounced at location c, j, k. The missing data at a'–i' and bounced data at c', j', k' are imputed to complete and smooth data by the Patch Program

**Fig. 13:**
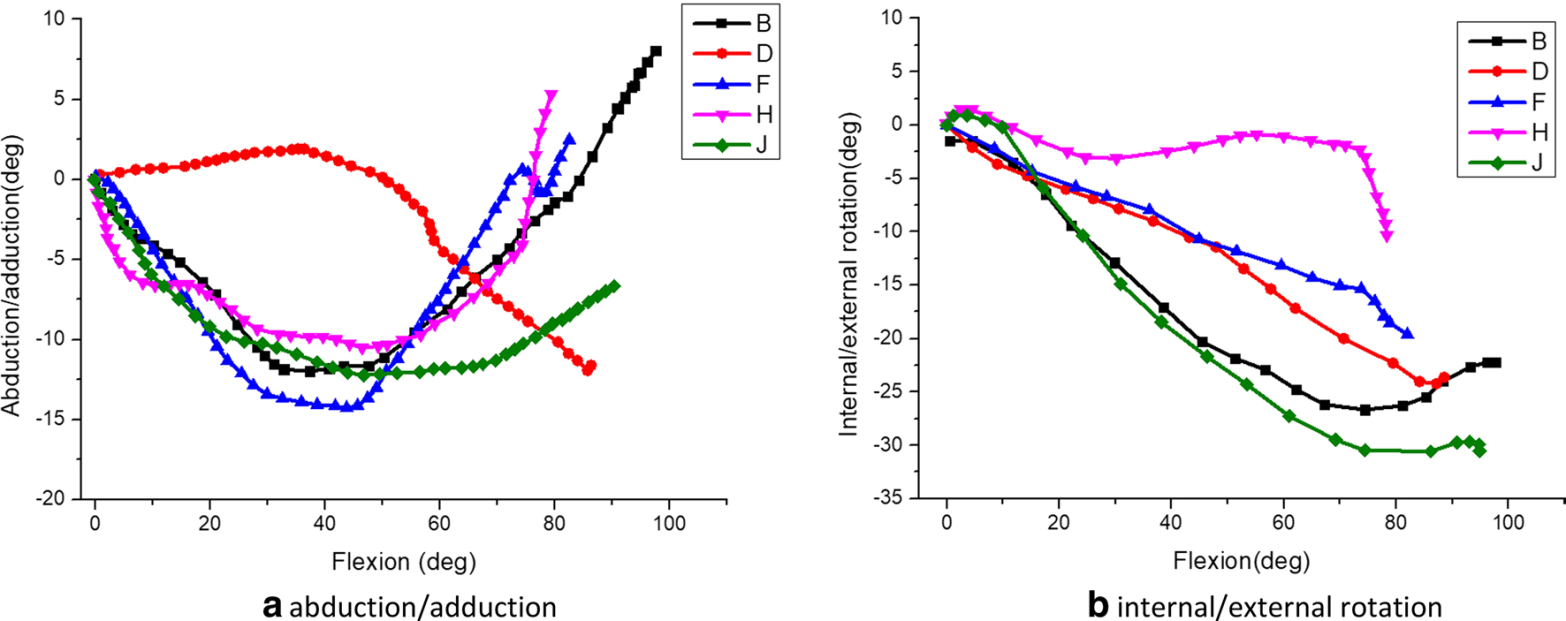
The relative rotation motion of femur vs tibia under different flexion during squat. *Note*: B/D/F/H/J respectively represent the motion data of internal/external rotation and adduction/abduction of five different volunteers in this study. 5 of 29 volunteer are randomly selected for processing avoiding messy figure

**Fig. 14:**
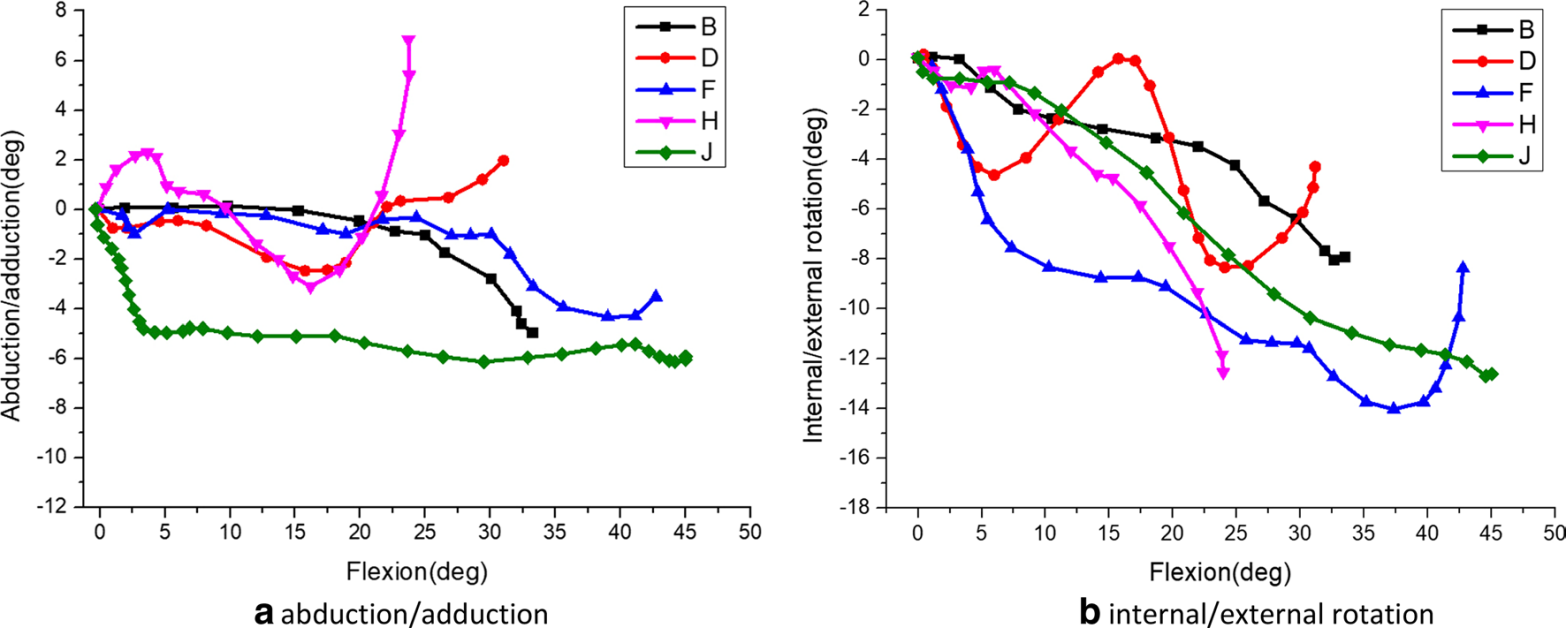
The relative rotation motion of femur vs tibia under different flexion during stairs climbing. *Note*: B/D/F/H/J respectively represent the motion data of internal/external rotation and adduction/abduction of five different volunteers in this study

The method described in this paper is applied to capture and measure the subjects' motion and process the data. Firstly, the 3D coordinate data of markers were obtained: if the origin data were intact, as shown in Fig. 8, then it can be processed directly by the universal (main) data processing program for obtaining the relative motion curve. If there are missing and noise data in the data, then the Patch Program has to be used to do the pre-processing the pre-patching data can be obtained as shown in Fig. 12a, and the post-patching data are shown in Fig. 12b, finally the same universal (main) data processing program was used to get the results as examples shown in Figs. 13 and 14.

Figure 12 shows the motion curve of three-dimensional coordinates that have been processed by the MATLAB Patch Program before and after. It is the three-dimensional coordinates of the human thigh (Marker 5–8) and calf (Marker 9–12) in the X, Y, and Z directions when a healthy subject squats. As shown in this figure, the data of Marker 5 on the subject's thigh were missing in the 370–400th frame in the three directions of X, Y, and Z (as shown in Fig. 12a at location a, f, and i); the data of Marker 6 were missing in frames 105–150, frames 152–155 and frames 190–215 in the three directions of X, Y, and Z (as shown in Fig. 12a at b, d, e, g, h); the data of Marker 7 and Marker 8 are relatively continuous. On the subjects' calf, the data of Marker 9–12 is relatively continuous as a whole, excepting for the data beating of individual frames (as shown in Fig. 12a at j, k). After being processed by the Patch Program in MATLAB in this paper, as shown in Fig. 12b, the missing and abnormally beating intervals have been reasonably interpolated, so as to obtain relatively complete motion curve data after reasonable interpolation.

After getting the patched data, the MATLAB main program was used to obtain the flexion/extension, adduction/abduction, and internal/external rotation data of the knee joint femur relative to the tibia during the movement of the lower limbs. As shown in Fig. 13a, b, respectively, the x-coordinate is knee flexion angle, and the y-coordinate is adduction-abduction and internal–external rotation. Figure 13 shows the adduction/abduction and internal/external rotation of the knee joint femur relative to the tibia at different flexion angles during squatting for five healthy subjects. During the squatting, the femur was abducted relative to the tibia from 0 degrees to about 40 degrees; from 40 degrees flexion on, the femur was adducting relative to the tibia until the knee reached the maximum flexion of 100 degrees. As the knee flexion angle deepened, the femur was abducted firstly and then adducted relative to the tibia, and the femur continued to rotate externally relative to the tibia. It can be seen from the figure that the maximum flexion angle of the knee joint in the squat was between 79 and 98 degrees in this experiment.

Figure 14 shows the adduction/abduction and internal/external rotation of the femur relative to the tibia during stairs climbing of five healthy subjects. It can be seen from the figure that the maximum flexion angle were within 23–44 degrees. Due to the higher mobility of the knee joint on the anterior–posterior axis, as the knee flexion increasing, there are relatively big difference in adduction/abduction, abducted firstly and then adducted overall. In the process of climbing the stairs, the femur continued to rotate externally relative to the tibia as a whole. However, the femur was rotated internally at the end of knee flexion in some subjects.

## Discussion

In this paper, a universal data processing program has also been developed for obtaining the relative movement of various components of the human body. A novel Patch Program of this paper has been developed, which can make reasonable compensation for missing and noise signals to obtain more complete motion data. At the same time, the motion capture measurement of the squat and stairs-climbing of the subjects was carried out, and the relative movements of tibiofemoral joint were analyzed as an example for the novel program’s applying.

### Verification and analysis

**Fig. 15:**
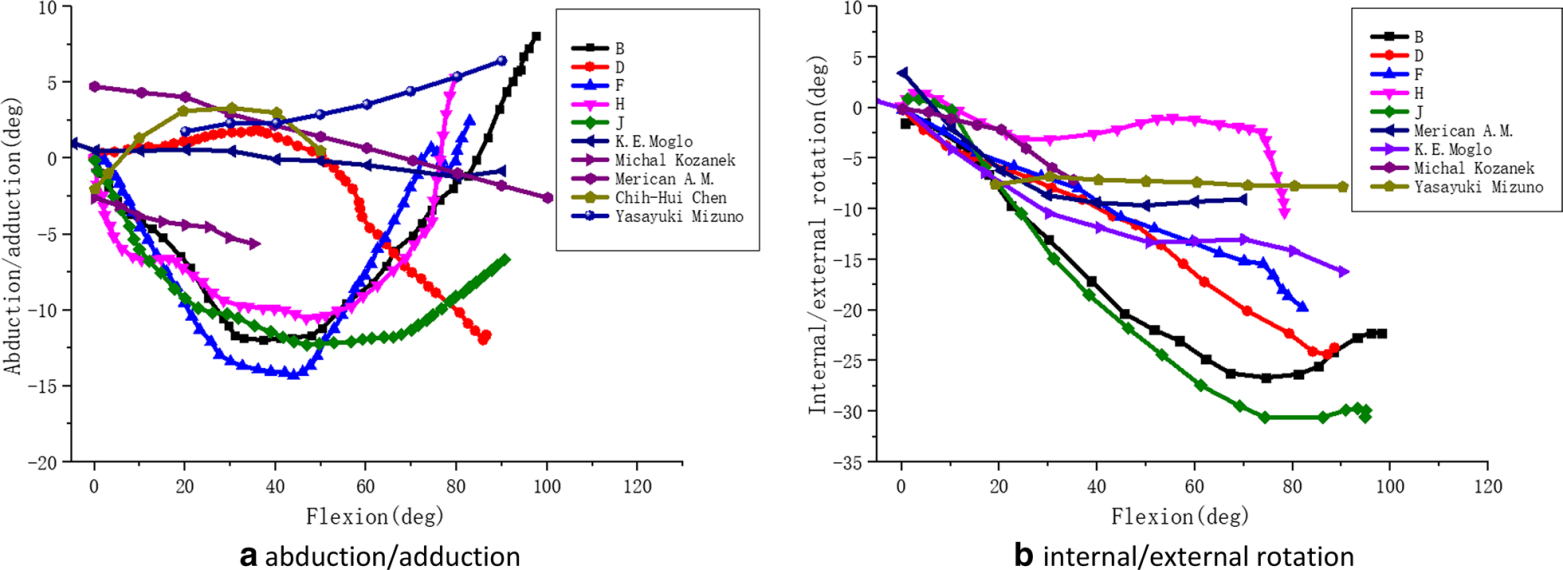
The comparative curves of adduction/abduction and internal/external rotation during squatting in this paper and other researches. *Note*: B/D/F/H/J respectively represent the motion data of internal/external rotation and adduction/abduction of five different volunteers in this study. The motion data of internal/external rotation and adduction/abduction of other researchers, which were compared with the data in this paper, were taken from the Moglo [41], Merican and Amis [42], Chen [43], Kozanek [44] and Mizuno [45]

**Fig. 16:**
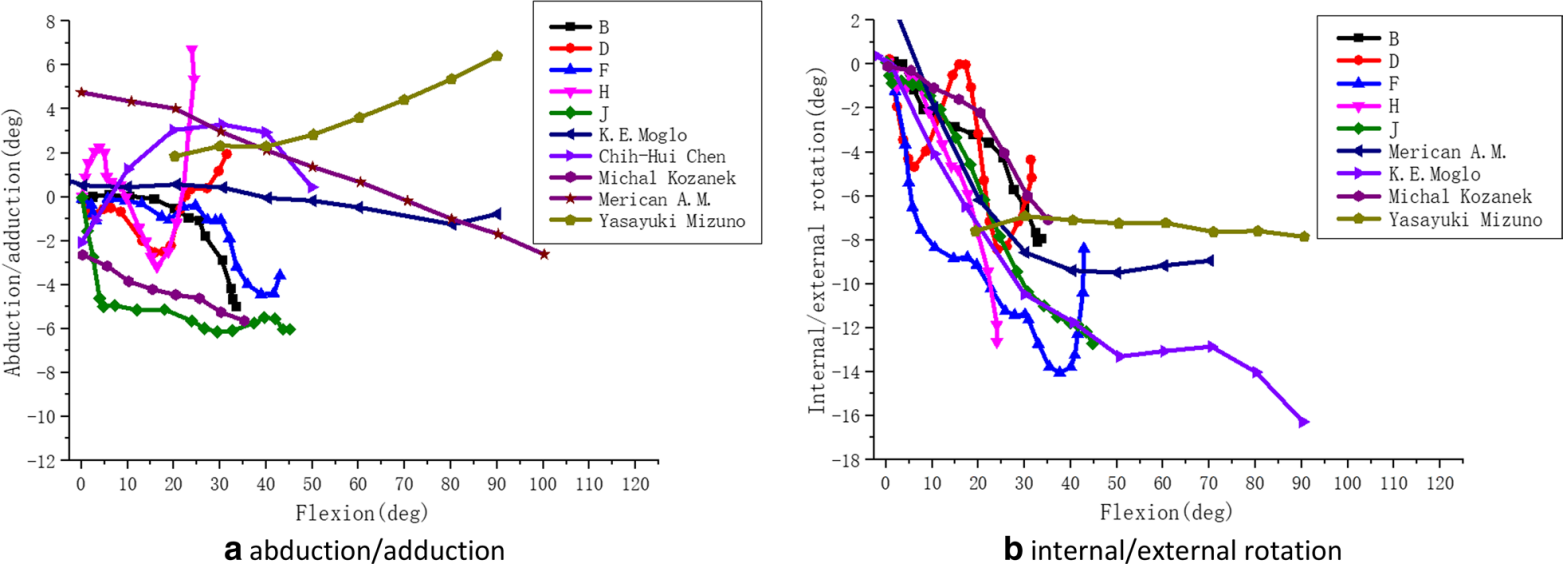
The comparative curves of adduction/abduction and internal/external rotation during climbing stairs in this paper and other researches. *Note*: B/D/F/H/J respectively represent the motion data of internal/external rotation and adduction/abduction of five different volunteers in this study. The motion data of internal/external rotation and adduction/abduction of other researchers, which were compared with the data in this paper, were taken from the Moglo [41], Merican and Amis [42], Chen [43], Kozanek [44] and Mizuno [45]

Accordingly, the results of this paper were compared with those of other researches (as shown in Figs. 15, 16).

As shown in Fig. 15a, for the adduction/abduction of the knee joint in the squat, Moglo et al. [41] showed that the femur was abducted relative to the tibia from − 10 to 0 degrees flexion and adducted from 0 to 80 degrees flexion. The results of Merican and Amis [42] showed that the femur was abducted firstly and then adducted relative to the tibia. The research in this paper also showed that the femur was abducted firstly, and the femur was adducted relative to the tibia at 40 degrees flexion. However, Chen et al. [43] showed that the femur was adducted and then abducted relative to the tibia, Kozanek et al. [44] have shown that the femur was abducted continuously relative to the tibia, while Mizuno et al. [45] have shown that the femur was adducted continuously relative to the tibia. The scope of adduction-abduction angle obtained in this paper was largely consistent with the comparison of other literatures. In summary, for different researchers and different test objects, the results of adduction and abduction in the process of knee flexion are different, which may be related to individual diversity.

As shown in Fig. 15b, for the internal–external rotation of the knee joint in the squat, the results of this paper shows that the femur was rotated externally relative to the tibia when the knee flexion increased, and the femur was rotated internally relative to the tibia around 70 degrees flexion of the knee joint. The results of Merican and Amis [42] also had the similar changes in the knee flexion. The result of Merican and Amis [42] indicated that the femur was changed from external rotation to internal rotation at about 70 degrees. The results of this paper indicated that the femur was generally in external rotation relative to the tibia, which was generally consistent with the results of Moglo et al. [41], Kozanek et al. [44] and Mizuno et al. [45]. In the research of Schmitz et al. [11], the squat (the flexion angle of the knee joint to 60 degrees) of 15 healthy people was measured by the motion capture system, and the data was processed by Visual 3D software. The results showed that in a squat cycle, from the beginning to the flexion angle of the knee joint reaching the maximum, the knee joint was always in external rotation. This was consistent with the trend of movement in this squat measurement.

As shown in Fig. 16a, for the adduction/abduction of the knee joint during the stairs-climbing, five subjects’ data was collected in this paper, one of them continued to abducted relative to tibia with the flexion of the knee joint, which was consistent with the results of Kozanek et al. [44]. Two of the five subjects in this paper abducted firstly, and then from 17 degrees flexion on, the femur was adducting relative to the tibia until the knee reached the maximum flexion. The results of Moglo et al. [41] and Merican and Amis [42] et al. showed that the femur was abducted firstly and then adducted relative to the tibia, which was consistent with the results in this paper. However, a few researchers had different results, for example the results of Chen et al. [43] showed that the femur was adducted firstly and then abducted relative to the tibia, and the results of Mizuno et al. [45] have shown that the femur was adducted continuously relative to the tibia. All in all, the above results indicated that adduction-abduction of the knee joint was unstable.

As shown in Fig. 16b, for the internal/external rotation of the knee joint during the stairs climbing, five subjects’ data were collected in this paper, three of them continued to rotate externally relative to the tibia with the flexion of the knee joint, which was generally consistent with the results of Moglo et al. [41], Kozanek et al. [44] and Mizuno et al. [45]. One of the five subjects in this paper was rotated interally relative to the tibia in the late stage of knee joint flexion, which was generally consistent with the results of Merican and Amis [42]. Besides, Riener [13] measured the upstairs movements of 10 normal people on the steps with different slopes, and analyzed the joint movement of the lower limbs. The results showed that the joint angle was significantly related to the step slope, and the larger the step slope was, the greater the maximum flexion angle of the knee joint during movements was. Tang Gang et al. [16] used a staircase with 160 mm in height to analyze the changes of the lower limb joint angle of healthy volunteers during the stairs climbing. It was pointed out that the maximum knee flexion range was 91.4 degrees, and the adduction-abduction range was − 3.2 to 8.9 degrees, and the internal–external rotation range was 20.5 degrees. The height of the stair used in this paper was 100 mm, and the greatest knee flexion angle during stairs climbing was 44 degrees. Meantime, the maximal range of the knee joint adduction-abduction was − 6 to 6.8 degrees, and the maximum angle of internal–external rotation was 14 degrees, which was in line with the research results in literature [13], but lower than that in literature [16]. It showed that adduction-abduction and internal–external rotation angle were within a reasonable range, and indicates the reliability and rationality of the experimental results in this paper.

Table 1 presents the comparison of the cited previous works based on follows: the type of techniques used, preprocessing techniques, range of flexion, abduction–adduction, internal–external rotation, the advantages and disadvantages offered.

**Table 1:** Comparison between this paper and other literatures

Name	Tech-used	Pre-process Techs	flexion	Abdu-addu	In-external rotation	Advantage	Disadvantage
Moglo [41]	Non-linear 3D finite element model	Meshing, Definition of material properties	− 5° to 90°	− 1° to 1°	− 16° to 0.5°	Avoiding the simultaneous consideration of large rotations about different axes on a single body	Caution should be exercised while extending the findings to the knee joint in living subjects in presence of different loads
Chen [43]	Dual fluoroscopic and MR image technique	Create 3D computer models, image registration	0–50°	− 2° to 3°	/	Being accurate, non-invasive	The view of the two fluoroscopes is limited
Kozanek [44]	Dual fluoroscopic imaging system	Create 3D computer models, image registration	0–40°	− 6° to 2.5°	− 7.5° to 0	Being accurate, non-invasive and no-requiring placement of external devices or markers on the knee	The relatively slow walking speed (0.67 m/s), the view of the two fluoroscopes is limited
Mizuno et al. [45]	The dynamic knee simulator	Standardized radiographic technique	0°–90°	1°–6°	− 7° to − 4°	Performing stable knee flexion under heavy muscle load	The cadaver model exactly no-representing anatomic Q-angle variations or surgical procedures modifying the Q-angle
Merican and Amis [42]	A Polaris optical tracking system	Thawed, kept moist with normal saline	0–70°	− 2° to 5°	− 10° to 4°	Being applied to the clinical on the effects of lateral retinacular releases to correct patellar mal-tracking	Elderly knees
Resultsof this paper	MATLAB	Pre-program and patch program	(0°–90°)*	(− 14° to 7°)*	(− 30° to 2°)*	The patch program developed in MATLAB making reasonable compensation for the undetectable points	Data preprocessing is not as fast as commercial software
			(0–45°)**	(− 6° to 7°)**	(− 14° to 0°)**		

1. ‘Tech-Used’ is short for ‘Technique Used’

2. ‘Pre-process’ is short for ‘Pre-processing Technique’

3. ‘flexion’ is short for ‘Range of flexion’

4. ‘Abdu-addu’ is short for ‘Abduction–adduction’

5. ‘*’ means ‘squatting’, ‘**’ means ‘Climbing stair’

### Limitation analysis of verification experiment

For the purpose of further comparation research, in this experiment, the TKA-After patients (Having the TKA operation within a short time) were conducted the same measurement. So considering the motion function difference between healthy and patients one, the height of stair steps was required to be lower than normal. Besides, RSA (Roentgen Stereophotogrammetric Analysis) and high-speed photographic experimental measurements were also synchronously carried out in this experiment to perform the contrastive analysis of the data. Due to the limited shooting space of experimental equipment and the space limitations caused by the simultaneous measurement of multiple systems, the volunteers' motion range was relatively small and the shooting time was limited within 5 s. Based on the above two main reasons, the maximum flexion of knee joint in the stair climbing and squatting experiment in this paper was relatively small.

This experiment calibrates the anatomical feature points under the guidance of a professional orthopedist. Multiple calibrations were required prior to formal testing to ensure accurate position of the anatomical feature points. Volunteers were required training for actions to enable them to perform as specified. In the formal experiment, each action was measured multiple times, and then the normal behavior of the most normal state was selected for analysis. However, the method described in this paper also has some limitations, for example, the motion capture measurement of human lower extremity motion and the calculation of kinematic parameters of the knee joint need to use the seven anatomical feature points to establish the coordinate system of the femur and tibia. Therefore, there is certain influence on the experimental results causing by the determination of the anatomical feature point position.

### Comparative analysis of processing methods

At present, there are a variety of methods for the processing of kinematics data measured by motion capture system and special software for data processing, including the de-noise processing of data, the matching of scattered data and the repair of missing points. Sheng [46] proposed a data processing algorithm based on the piecewise linear model of modules, which can effectively carry out global hierarchical prediction and tracking of 3D motion data modules, and module-based de-noise processing of noise data, and put forward piecewise Newton interpolation operation based on missing motion data to make reasonable supplement. In this paper, cubic spline interpolation function is used to repair the interpolation program. The interpolated curve of this method is second-order continuous and differentiable, and the interpolation operation method of piecewise Newton interpolation has high accuracy and smooth curve transition. Alonso et al. [47] proposed to use the inverse dynamics model of human skeleton for motion data processing, where the complement algorithm based on rigid body requires that there should not be too many defects in the rigid body. For example, for the four waist points, matching complement points can be obtained only when one point is missing, which is the same idea as the repair data in this paper. When one of four marker points missed, it can be repaired; when two or more marker points missed, it cannot be repaired, so it needs to be abandoned. Liu [48] et al. used piecewise linear PCA technology to estimate the missing points and introduced statistical theory into motion data processing for statistical analysis. In terms of motion data processing software, as mentioned in literature [11], Vicon optical infrared motion capture system [49], was used to measure lower limb motion by motion capture system, and Visual3D commercial software was used for data processing, which was relatively expensive. At the initial step of data processing in commercial software, every testee’s columnar motion model needs to be constructed manually by using the ‘BodyBuilder’ module of commercial software, which need costing much time and energy. After this initial manual process, although these software can automatically analyze and report motion capture data, if there are some occluded points and points being not captured during the movement measurement, the data need to be processed manually one by one in these software. Due to the data was large sample size, it also consumes a lot of time and energy. However, in this paper, the developed MATLAB program written by the authors can be used to batch processing motion capture data while avoiding manual modeling one by one. Meantime a novel Patch Program also had been developed to repair missing or noise data. The local coordinate systems of tibia and femur can be established by using bone markers in the program. At last, according to the coordinate transformation, the kinematic parameters of knee femur relative to tibia in the movement of human lower limbs were quickly calculated, and the spatial coordinate curve of tibia and femur in the movement process also can be obtained. Therefore, human motion can be processed quickly by batch processing, and the program can be modified to meet different special requirements of motion function analysis. In the future, more accurate kinematics data will be obtained by increasing the sample size and continuously improving the program by this paper’s authors.

### The hypothesis of the developed method

The RSA (Roentgen Stereophotogrammetric Analysis), NDI (motion capture) and high speed stereo radiography (HSSA) were synchronously used to measure the motion of the human knee joint during three movement types (squatting, walking, and climbing stairs), and further mechanism analysis of tibiofemoral in three type movements will be made furtherly. At the same time, the error analysis of micro motion of marker point on skin surface will be considered and analyzed for establishing the error model of motion capture, so as to reduce the motion capture measuring error, and the accuracy of motion measurement can be improved.

### Analysis of total advantages and disadvantages

In the calibration period of motion capture, marker should be established for each volunteer. However, the method in this paper is convenient and fast because of insteading of manually establishing marker one by one on the model in the post-processing of data in business software. A novel MATLAB program had been developed for batch processing, and the developed Patch Program in this paper can make reasonable compensation for the missing, noise and undetectable points of motion capture. Compared with the methods in other literatures [50, 51], a Patch Program was added in this paper, as shown in Fig. 12. The patch program can make reasonable compensation for the missing and abnormally beating intervals during the motion capture process, so as to obtain relatively complete motion curve data after reasonable interpolation. Meantime a universal data processing program has also been developed for obtaining the relative movement of various components of the human body, and the program can be modified for detail special analysis. In addition, the research has many aspects in medical applications: (1) through gait measurement to find out whether the human body is sick. (2) It can provide correct motion parameters for rehabilitation treatment and rehabilitation medical design by measuring different human bodies and different movements of the human body. (3) Comparing the motion parameters (between pre–post surgical operation) to analyze the treatment effect of the operation and make a plan of further treatment. Of course, MATLAB has certain limitations to this analysis. MATLAB's pre-processing is not as fast as commercial software.

## Conclusions

Novel programs were developed in MATLAB software, and a set of methods was designed to process motion capture data in this paper. The results of other researchers are consistent with the movement trend of knee squatting and stairs climbing in this paper, which also verifies the validity and rationality of the data processing method and the developed programs in this paper. For the most of popular commercial software, models and markers for each volunteer should be established during the calibration and data pre-processing phase. After calibration, the program developed in this paper, which can directly and efficiently batch process data and avoid manual modeling one by one, is convenient and fast. In view of data missing and beating signals, a novel Patch Program was developed to make reasonable compensation, acquiring more complete motion data. At the same time, a universal data processing program has also been developed for obtaining the relative movement of various components of the human body, and the program can be modified for detail special analysis. These motion capture technologies can be used to judge whether the human body functions are abnormal, provide a reference for rehabilitation treatment and the design of rehabilitation equipment, and can evaluate the effect of pre–post-surgery.

## Figures and Tables

**Fig. 17:**
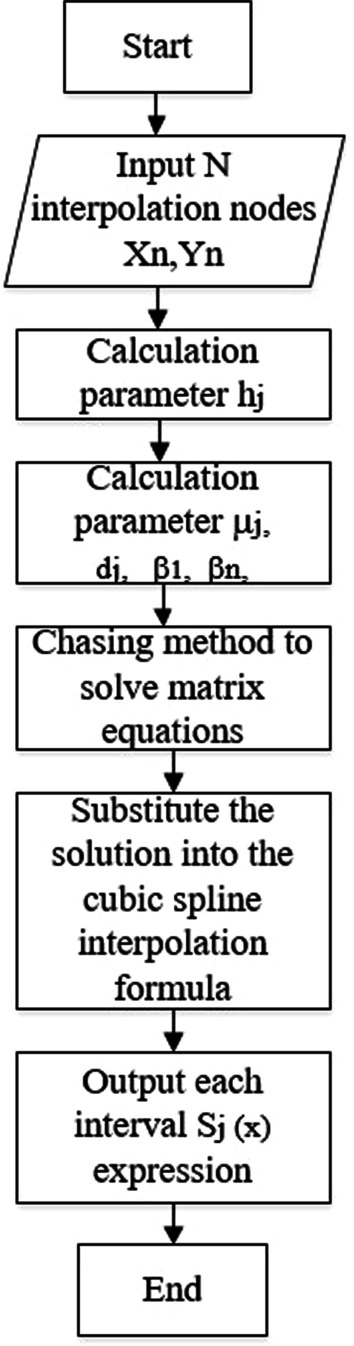
Block diagram for solving cubic spline interpolation function

## Data Availability

The datasets used and/or analyzed during the current study are available from the corresponding author on reasonable request.

## Appendix 1: Cubic spline interpolation function principle

The block diagram for solving cubic spline interpolation function is shown in Fig. 17. The cubic spline interpolation function can be solved according to definitions and boundary conditions. Given some data point correspondence function values in the interval [a, b], $\left\{ {x_{1} ,x_{2} , \ldots ,x_{n} } \right\}$, corresponding function value $\left\{ {y_{1} ,y_{2} , \ldots ,y_{n} } \right\}$, S(x) is a cubic spline interpolation function, which satisfies $S(x_{j} ) = y_{j} (j = 1,2, \ldots ,n)$ and has a second-order continuous derivative on [a, b]. S(x) determines a cubic polynomial on each subinterval $[x_{j} ,x_{j + 1} ]$, known $S^{{\prime\prime}} (x_{j} ) = M_{j} ,S^{{\prime\prime}} (x_{j + 1}^{{}} ) = M_{j + 1}$, $h_{j} = x_{j + 1} - x_{j} (j = 1,2,\ldots,n - 1)$, then

3 $$S(x) = \frac{{(x_{j + 1} - x)^{3} }}{{6h_{j} }}M_{j} + \frac{{(x - x_{j} )^{3} }}{{6h_{j} }}M_{j + 1} + \left( {y_{j} - \frac{{M_{j} }}{6}h_{j}^{2} } \right)\frac{{x_{j + 1} - x}}{{h_{j} }} + \left( {y_{j + 1} - \frac{{M_{j + 1} }}{6}h_{j}^{2} } \right)\frac{{x - x_{j} }}{{h_{j} }}$$

The system of equations in matrix form was solved finally according to known conditions.

4 $$\begin{aligned} & \left[ {\begin{array}{*{20}c} 2 & 1 & {} & {} & {} \\ {\mu_{2} } & 2 & {1 - \mu_{2} } & {} & {} \\ {} & . & . & . & {} \\ {} & {} & {\mu_{n - 1} } & 2 & {1 - \mu_{n - 1} } \\ {} & {} & {} & 1 & 2 \\ \end{array} } \right]\left[ {\begin{array}{*{20}c} {M_{1} } \\ {M_{2} } \\ . \\ {M_{n - 1} } \\ {M_{n} } \\ \end{array} } \right] = \left[ {\begin{array}{*{20}c} {\beta_{1} } \\ {d_{2} } \\ . \\ {d_{n - 1} } \\ {\beta_{n} } \\ \end{array} } \right] \\ & \mu_{j} = \frac{{h_{j - 1} }}{{h_{j - 1} + h_{j} }},\quad d_{j} = 6\left( {\frac{{y_{j + 1} - y_{j} }}{{h_{j} }} - \frac{{y_{j} - y_{j - 1} }}{{h_{j - 1} }}} \right)\frac{1}{{h_{j - 1} + h_{j} }}, \\ & \beta_{1} = \frac{6}{{h_{1} }}\left( {\frac{{y_{2} - y_{1} }}{{h_{1} }} - y_{1}^{{\prime}} } \right),\quad \beta_{n} = \frac{6}{{h_{n - 1} }}\left( {y_{n}^{{\prime}} - \frac{{y_{n} - y_{n - 1} }}{{h_{n - 1} }}} \right), \\ \end{aligned}$$

The coefficient matrix of Eq. (4) is a three-diagonal matrix and a diagonally dominant matrix, so there is a unique solution. The interpolation function S(x) on [a, b] is obtained by taking the solution into Eq. (3).

## Appendix 2: The function principle of transformation matrix [31]

In order to obtain the estimation of motion parameters of a rigid object using 3-D point correspondences and the determination of the relative attitude of a rigid object with respect to a reference, there are 2 sets of three-dimensional points $\{ p_{i} \}$ and $\{ p_{i}^{{\prime}} \}$ on the rigid body, i = 1, 2, …, N, N is the number of markers, and $\{ p_{i} \}$ and $\{ p_{i}^{{\prime}} \}$ are 3 × 1 column matrix, then there is $p_{i}^{{\prime}} = Rp_{i} + T$, where R is the $3 \times 3$ rotation matrix, and T is the $3 \times 1$ translation vector of the column matrix.

5 $$p_{i}^{{\prime}} = Rp_{i} + T$$

- *Step 1*: From $\{ p_{i} \}$, $\{ p_{i}^{{\prime}} \}$ calculate p, p′; and then $q_{i}$, $q_{i}^{{\prime}}$
  6 $$p = \frac{1}{N}\sum\limits_{i = 1}^{N} {p_{i} } \quad p^{{\prime}} = \frac{1}{N}\sum\limits_{i = 1}^{N} {p_{i}^{{\prime}} }$$
  7 $$q_{i} = p_{i} - p\quad q_{i}^{{\prime}} = p_{i}^{{\prime}} - p^{{\prime}}$$
- *Step 2*: Calculate $3 \times 3$ matrix
  8 $$H = \sum\limits_{i = 1}^{N} {q_{i} q_{i}^{{\prime}t} }$$
  where the superscript *t* denotes matrix transposition.
- *Step 3*: Find the SVD of H.
  9 $$H = U\Lambda V^{t}$$
- *Step 4*: Calculate
  10 $$X = VU^{t}$$
- *Step 5*: Calculate, det (*x*), the determinant of X.
- If det(x) =  + 1, then $\mathop R\limits^{ \wedge } = X$.
- If det(x) = − 1, the algorithm fails. (This case usually does not occur.)

According to the above, we get the T, R conversion matrix. According to this formula $p_{i}^{{\prime}} = Rp_{i} + T$, the change of the relative motion posture of the object can be obtained.

## Abbreviations

TKA: Total knee arthroplasty
3D: Three-dimensional
